# Time in Range as a Marker of Concurrent Glycemic and Lipid Improvement in Adults with Type 2 Diabetes: A Real-World Retrospective Pre–Post Study

**DOI:** 10.3390/life16101667

**Published:** 2026-10-05

**Authors:** Mi-Joon Lee, Bum-Jeun Seo, Jae-Hyoung Cho

**Affiliations:** 1 Department of Medical Information, Kongju National University, 56 Gongjudaehak-ro, Gongju-si 32588, Republic of Korea; mijoon1004@kongju.ac.kr; 2 Department of Internal Medicine, Seoul St. Mary’s Hospital, College of Medicine, The Catholic University of Korea, Seoul 06591, Republic of Korea; drhopper@catholic.ac.kr

**Keywords:** type 2 diabetes mellitus, continuous glucose monitoring, time in range, glycemic control, glycated hemoglobin, lipid metabolism

## Abstract

This study aims to evaluate whether continuous glucose monitoring (CGM)-derived time in range (TIR) attainment is associated with concurrent improvements in glycemic control and lipid profiles among adults with type 2 diabetes mellitus (T2DM). In this retrospective, single-arm pre–post study, 542 adults with T2DM who underwent intermittently scanned CGM for 12 weeks were analyzed. Glycated hemoglobin (HbA1c), fasting plasma glucose (FPG), total cholesterol, triglycerides, high-density lipoprotein cholesterol (HDL-C), and low-density lipoprotein cholesterol (LDL-C) were compared before and after CGM use. Participants were stratified by TIR into low (<50%), intermediate (50–70%), and target (>70%) groups. Overall, HbA1c decreased from 8.12% to 7.49% and FPG from 153.35 to 136.70 mg/dL (both *p* < 0.001). Significant improvements were also observed in total cholesterol, triglycerides, HDL-C, and LDL-C. Glycemic improvement occurred across all TIR strata; however, significant improvements in all lipid parameters were observed only in participants achieving TIR >70%. In this group, total cholesterol decreased by 4.23 mg/dL, triglycerides by 11.12 mg/dL, and LDL-C by 4.48 mg/dL, and HDL-C increased by 1.34 mg/dL. These findings suggest that achieving the consensus TIR target of >70% may be associated with broader cardiometabolic benefits beyond glycemic improvement alone. TIR may therefore serve as a clinically useful complementary target to HbA1c in routine diabetes management.

## 1. Introduction

Glycated hemoglobin (HbA1c) has served for decades as the reference standard for assessing long-term glycemic control and as the principal treatment target in diabetes care [1]. Its central limitation, however, is that it summarizes glycemia as a single time-averaged value. Two patients with an identical HbA1c may follow entirely different glucose trajectories: one may remain close to the target range throughout the day, while the other may oscillate repeatedly between hyperglycemia and hypoglycemia. HbA1c is therefore unable to characterize the direction, amplitude, and frequency of glucose excursions, all of which are increasingly regarded as clinically meaningful dimensions of glycemic control [2].

Continuous glucose monitoring (CGM) has fundamentally changed how glycemic control can be described. By generating glucose values at intervals of minutes, CGM permits quantification of the proportion of time spent within, above, and below a defined target range, giving rise to a family of time-based glycemic metrics. Foremost among these is time in range (TIR), defined as the percentage of time with sensor glucose between 70 and 180 mg/dL. In 2019, the International Consensus on Time in Range proposed a standardized set of ten core CGM metrics, recommending TIR > 70%, time below range (TBR) < 4%, and a coefficient of variation (CV) < 36% as targets for most adults with diabetes [3].

Glycemic variability—the magnitude and frequency with which glucose deviates from its mean over a given period—is conceptually distinct from mean glycemia and is not fully captured by HbA1c [4]. Although some degree of variability occurs in individuals without diabetes, it is markedly amplified in diabetes. Mechanistically, repeated oscillation between hyperglycemia and hypoglycemia is thought to promote oxidative stress, endothelial dysfunction, and low-grade inflammation to a greater extent than sustained hyperglycemia of equivalent mean magnitude [5], and glycemic variability has been associated with mortality in critically ill populations [6].

A growing body of evidence links TIR specifically to diabetes-related complications. Lower TIR has been associated with diabetic retinopathy [7], increased carotid intima-media thickness [8], albuminuria [9], and painful diabetic polyneuropathy [10] in type 2 diabetes, and a prospective cohort study reported graded increases in all-cause and cardiovascular mortality across decreasing TIR strata [11]. These observations are complemented by evidence from the ACCORD trial, in which intensive glucose lowering aimed at near-normal HbA1c was associated with increased cardiovascular mortality [12], and by the subsequent finding that long-term HbA1c variability predicted mortality more strongly than mean HbA1c itself [13]. Together, these data support a paradigm shift in which HbA1c alone is regarded as insufficient for risk stratification and complementary time-based metrics are required.

Concurrently, clinical adoption of CGM has accelerated, driven by improvements in sensor accuracy, ease of use, and expanding national reimbursement, including in the Republic of Korea [14,15]. Current international guidance from the American Diabetes Association and the European Association for the Study of Diabetes advocates person-centered care in which TIR, TBR, and glycemic variability are evaluated alongside HbA1c [16], and meta-analytic evidence indicates that CGM improves HbA1c, increases TIR, and reduces hypoglycemia [17].

Despite this progress, most CGM literature has focused on glycemic endpoints, and relatively little real-world evidence addresses whether attainment of the recommended TIR threshold is accompanied by improvement in other cardiometabolic risk factors, particularly the lipid profile. Given that dyslipidemia is a principal driver of macro-vascular risk in type 2 diabetes, clarifying this relationship has direct clinical relevance [18].

This study therefore examined, in a large real-world cohort of adults with type 2 diabetes undergoing CGM in routine tertiary care, (i) the distribution of CGM-derived glycemic metrics; (ii) changes in glycemic and lipid parameters between the pre- and post-CGM periods; (iii) whether the magnitude of change in triglycerides differed according to sociodemographic and health-related characteristics; and (iv) whether the pattern of glycemic and lipid change differed according to the level of TIR attainment.

## 2. Materials and Methods

### 2.1. Study Design

This was a single-center, retrospective, single-arm pre–post study using routinely collected electronic medical record (EMR) data [19]. Each patient served as his or her own control; no concurrent comparison group was available. The exposure period was defined as the interval during which the CGM sensor was worn, and glycemic and lipid parameters measured at the visit immediately preceding sensor application (baseline) were compared with those measured at the first subsequent follow-up visit (post-CGM) (Table 1).

### 2.2. Study Subjects

This retrospective EMR-based study included patients with T2DM who received CGM between July 2018 and October 2024. A total of 1000 patient records were extracted from the institutional electronic medical record database during the study period. Of these, 458 patients were excluded because complete paired baseline and follow-up clinical or laboratory data required for the pre–post analysis were unavailable, leaving 542 participants in the final analytic cohort. Eligible participants were adults aged 20 years or older, had a physician-confirmed diagnosis of T2DM, received a CGM prescription, and agreed to the use of their de-identified clinical data for research purposes.

### 2.3. Continuous Glucose Monitoring

Glucose monitoring was performed using the FreeStyle Libre intermittently scanned CGM system (Abbott Diabetes Care, Witney, UK) [20]. The sensor is factory-calibrated and requires no confirmatory capillary blood glucose calibration, and each device can be worn for up to 14 days. The system comprises a subcutaneous sensor that measures interstitial fluid glucose, an integrated transmitter, and a reader or smartphone application that displays and stores the transmitted values.

The following CGM-derived metrics were extracted from the manufacturer’s reporting software [21]: mean sensor glucose; glucose management indicator (GMI), an estimate of laboratory HbA1c calculated from mean sensor glucose; CV, expressed as the ratio of the standard deviation of sensor glucose to mean sensor glucose; sensor active time; time above range level 2 (TAR2, >250 mg/dL); time above range level 1 (TAR1, 181–250 mg/dL); TIR (70–180 mg/dL); time below range level 1 (TBR1, 54–69 mg/dL); and time below range level 2 (TBR2, <54 mg/dL) [3]. All metrics were computed over the full wear period (12 weeks). Because GMI is derived from sensor glucose rather than from a blood sample, it may differ from concurrently measured laboratory HbA1c.

### 2.4. Laboratory Measurements

Laboratory assessments included glycated hemoglobin (HbA1c), fasting plasma glucose (FPG), total cholesterol, triglycerides, high-density lipoprotein (HDL) cholesterol, and low-density lipoprotein (LDL) cholesterol. Measurements were obtained during routine outpatient visits before CGM initiation and at follow-up after CGM use using the hospital’s standard laboratory procedures. Baseline laboratory measurements were defined as those obtained at the routine outpatient visit immediately preceding CGM initiation, whereas follow-up measurements were obtained at the first subsequent outpatient visit corresponding approximately to the 12-week follow-up period. Because this was a retrospective real-world study based on routine clinical practice, no predefined allowable time window or margin for laboratory sampling relative to CGM initiation or the intended 12-week follow-up was applied. Consequently, the exact timing of laboratory assessments could vary among participants according to their routine clinical visit schedules.

### 2.5. Classification of TIR Groups

The study subjects were stratified into three mutually exclusive groups according to the TIR value recorded over the CGM wear period: Low-TIR (<50%), Intermediate-TIR (50–70%), and Target-TIR (>70%). These thresholds were selected based on clinically relevant targets proposed in international consensus recommendations [3,22]. A TIR >70% is recommended as the primary treatment target for most non-pregnant adults with diabetes, whereas a TIR >50% has been proposed as a less stringent, individualized target for older adults or individuals at high risk of hypoglycemia. Accordingly, the 50% cutoff was used in the present study as a clinically meaningful threshold for stratification rather than as a universal therapeutic target for the entire study population. The three groups were therefore defined as follows:

- Low-TIR group: TIR < 50%;
- Intermediate-TIR group: TIR 50–70%;
- Target-TIR group: TIR > 70% (consensus target attained).

### 2.6. Ethical Considerations for Participants

To ensure the protection of personal information and data security, patient data obtained from the electronic medical record (EMR) system of individuals treated for diabetes were de-identified. All personal identifiers were removed and replaced with placeholder values to maintain anonymity. The study was conducted in accordance with the Declaration of Helsinki and approved by the Institutional Review Board of B University (IRB No. 2-7008132-A-N-01. 25-11).

### 2.7. Data Collection and Statistical Analysis

Data processing and statistical testing were performed using IBM SPSS Statistics for Windows, Version 27.0 (IBM Corp., Armonk, NY, USA). Descriptive data for continuous variables are presented as mean ± standard deviation (SD), whereas categorical variables are presented as frequency counts and percentages (*n*, %). Pre- and post-intervention differences in primary glycemic and lipid variables were evaluated using paired-sample t-tests across the total cohort and within individual TIR strata. Subgroup comparisons of triglyceride changes across demographic and lifestyle categories were analyzed using independent two-sample t-tests or one-way Analysis of Variance (ANOVA) complemented by Scheffé post hoc test for multiple comparisons. The statistical significance was set at *p*-value <0.05 [23].

## 3. Results

### 3.1. Baseline Characteristics of Study Subjects

Table 2 presents the baseline demographic and health-related characteristics of subjects. The study population comprised 542 adult patients with T2DM. Male participants constituted 63.5% (*n* = 344) of the cohort, while female participants accounted for 36.5% (*n* = 198). The overall mean age was 60.41 ± 11.17 years. Middle-aged adults aged 40–64 years represented the largest demographic proportion (56.3%, *n* = 305), followed by older adults aged over 65 years (39.6%, *n* = 215) and younger adults aged 18–39 years (4.1%, *n* = 22).

The mean baseline Body Mass Index (BMI) was 25.25 ± 3.72 kg/m^2^. Based on regional Asia-Pacific BMI categories, 50.6% (*n* = 274) of participants were classified as obese (BMI ≥ 25.0 kg/m^2^), 47.6% (*n* = 258) as normal weight (BMI 18.5–24.9 kg/m^2^), and 1.8% (*n* = 10) as underweight (BMI < 18.5 kg/m^2^). Assessment of lifestyle characteristics indicated that 80.3% (*n* = 435) were never smokers, 10.7% (*n* = 58) former smokers, and 9.0% (*n* = 49) current smokers. The proportion of non-drinkers in the cohort was 88.9% (*n* = 482).

### 3.2. Continuous Glucose Monitoring Metrics

The 12-week CGM profiles revealed a mean fasting glucose concentration of 167.99 ± 37.57 mg/dL and a calculated GMI of 7.47 ± 3.25%. The cohort exhibited a mean glucose coefficient of variation (%CV) of 29.93 ± 7.21%, which met the international glycemic stability benchmark of 36%. The mean rate of sensor active time was 84.93 ± 18.35%, satisfying consensus standards for data reliability.

The overall mean TIR (70–180 mg/dL) was 65.30 ± 21.87%, slightly below the recommended target benchmark of 70%. The proportion of time spent in level 1 hyperglycemia (TAR1; 181–250 mg/dL) was 23.01 ± 11.37%, while level 2 hyperglycemia (TAR2; >250 mg/dL) accounted for 10.85 ± 14.62%. Hypoglycemic exposure was low, with level 1 TBR (TBR1; 54–69 mg/dL) averaging 0.72 ± 2.14% and level 2 TBR (TBR2; <54 mg/dL) averaging 0.13 ± 1.60%, both adhering to clinical safety guidelines (<4% and <1%, respectively) [24] (Table 3).

### 3.3. Changes in Glycemic and Lipid Parameters Following CGM Use

In Table 4, following 12 weeks of CGM-guided management, the total cohort demonstrated statistically significant improvements in both glycemic control and serum lipid parameters. Mean HbA1c declined by 0.63%, dropping from 8.12 ± 1.39% at baseline to 7.49 ± 1.09% post-intervention (*p* < 0.001). Fasting plasma glucose decreased by 16.65 mg/dL, changing from 153.35 ± 58.01 mg/dL to 136.70 ± 41.31 mg/dL (*p* < 0.001).

Statistically significant favorable shifts were also observed across all circulating lipid fractions. Total cholesterol decreased by 3.17 mg/dL (150.11 ± 38.23 mg/dL to 146.94 ± 34.49 mg/dL; *p* = 0.018). Serum triglycerides declined by 9.80 mg/dL (144.19 ± 103.53 mg/dL to 134.39 ± 93.47 mg/dL; *p* = 0.011). HDL cholesterol increased by 0.85 mg/dL (46.38 ± 12.00 mg/dL to 47.23 ± 12.30 mg/dL; *p* = 0.004). LDL cholesterol decreased by 2.80 mg/dL (75.07 ± 30.39 mg/dL to 72.27 ± 26.72 mg/dL; *p* = 0.013).

### 3.4. Subgroup Analysis for the Triglyceride Change Stratified by Sociodemographic and Health-Related Characteristics

In Table 5, subgroup analysis revealed significant variations in triglyceride changes across demographic and anthropometric categories. Male participants achieved a statistically significant mean reduction in triglycerides of −16.27 ± 99.58 mg/dL, whereas female participants exhibited a slight mean increase of +1.46 ± 66.79 mg/dL (*p* = 0.014).

Age-stratified analysis demonstrated significant variance across age groups (*p* = 0.019). Young adults aged 18–39 years experienced a substantial triglyceride reduction of −61.82 ± 144.54 mg/dL, which was significantly greater than the reductions observed in middle-aged adults (−6.36 ± 93.92 mg/dL) and older adults (−9.35 ± 72.87 mg/dL). Post hoc Scheffé testing confirmed that triglyceride lowering in the 18–39 age group was significantly greater than in both older age brackets (*p* < 0.05).

Triglyceride responses also varied significantly across baseline BMI categories (*p* = 0.026). Normal-weight participants (BMI 18.5–24.9 kg/m^2^) achieved a mean triglyceride reduction of −17.05 ± 83.35 mg/dL, while obese individuals (BMI ≥ 25.0 kg/m^2^) experienced a mean reduction of −5.23 ± 93.81 mg/dL. In contrast, underweight participants (BMI < 18.5 kg/m^2^) exhibited an average triglyceride increase of +52.40 ± 90.74 mg/dL. Post hoc testing confirmed a statistically significant difference between normal-weight and underweight participants (*p* < 0.05). Differences in triglyceride changes across smoking status (*p* = 0.633) and alcohol consumption categories (*p* = 0.987) were not statistically significant.

### 3.5. Glycemic and Lipid Parameter Changes After 12-Week CGM Use Stratified by Time in Range (TIR) Achievement

To investigate whether metabolic improvements depend on specific TIR thresholds, patients were stratified into three achievement levels: low group (TIR < 50%), intermediate group (TIR 50–70%), and target group (TIR > 70%) (Table 6).

In the low group (TIR < 50%), CGM intervention yielded significant improvements in glycemic parameters. Mean HbA1c decreased from 9.27 ± 1.72% to 8.41 ± 1.25% (mean change −0.86, *p* < 0.001), and fasting glucose fell from 187.95 ± 79.33 mg/dL to 159.26 ± 62.04 mg/dL (mean change −28.69 mg/dL, *p* = 0.001). However, no statistically significant changes were observed in serum lipid parameters: total cholesterol (*p* = 0.904), triglycerides (*p* = 0.113), HDL cholesterol (*p* = 0.197), or LDL cholesterol (*p* = 0.718).

In the intermediate group (TIR 50–70%), glycemic markers improved significantly, with HbA1c decreasing from 8.26 ± 1.14% to 7.64 ± 0.93% (mean change −0.62, *p* < 0.001) and fasting glucose declining from 156.08 ± 57.01 mg/dL to 137.71 ± 35.46 mg/dL (mean change −18.37 mg/dL, *p* < 0.001). Similar to the low group, concurrent modifications in lipid parameters did not reach statistical significance: total cholesterol (*p* = 0.133), triglycerides (*p* = 0.560), HDL cholesterol (*p* = 0.826), and LDL cholesterol (*p* = 0.230).

In contrast, participants achieving the target TIR (> 70%) demonstrated statistically significant improvements across both glycemic parameters and all evaluated lipid fractions. Mean HbA1c decreased from 7.56 ± 1.01% to 7.02 ± 0.80% (mean change −0.54, *p* < 0.001), and fasting glucose dropped from 137.29 ± 38.55 mg/dL to 126.70 ± 28.20 mg/dL (mean change −10.59 mg/dL, *p* < 0.001). Concurrently, total cholesterol decreased from 149.90 ± 39.27 mg/dL to 145.67 ± 33.88 mg/dL (mean change −4.23 mg/dL, *p* = 0.020), triglycerides fell from 136.99 ± 106.35 mg/dL to 125.87 ± 99.25 mg/dL (mean change −11.12 mg/dL, *p* = 0.038), HDL cholesterol increased from 47.50 ± 11.55 mg/dL to 48.84 ± 11.88 mg/dL (mean change +1.34 mg/dL, *p* = 0.001), and LDL cholesterol declined from 74.77 ± 30.86 mg/dL to 70.29 ± 26.38 mg/dL (mean change −4.48 mg/dL, *p* = 0.004).

Although triglyceride levels decreased significantly within the TIR >70% group in the categorical analysis, analysis of TIR as a continuous variable did not demonstrate a clear linear or nonlinear relationship with triglyceride change (Supplementary Figure S1). Therefore, the present findings do not support a distinct therapeutic threshold at TIR >70%.

### 3.6. Effects of Time and TIR Group on Glycemic and Lipid Parameter Changes During 12-Week CGM Use

To formally compare changes in glycemic and lipid parameters across the TIR groups, repeated-measures ANOVA was performed to evaluate changes in glycemic and lipid parameters over 12 weeks according to TIR group (Supplementary Table S1). Significant time effects were observed for HbA1c (F = 123.820, *p* < 0.001), fasting glucose (F = 50.693, *p* < 0.001), triglycerides (F = 6.121, *p* = 0.014), and HDL cholesterol (F = 5.937, *p* = 0.015), indicating significant overall changes in these parameters during the study period. In contrast, the time effects for total cholesterol (F = 3.239, *p* = 0.072) and LDL cholesterol (F = 2.946, *p* = 0.087) were not statistically significant.

Significant main effects of TIR group were also observed for HbA1c (F = 126.112, *p* < 0.001), fasting glucose (F = 48.416, *p* < 0.001), triglycerides (F = 3.025, *p* = 0.049), and HDL cholesterol (F = 3.697, *p* = 0.025), whereas no significant between-group effects were observed for total cholesterol (*p* = 0.673) or LDL cholesterol (*p* = 0.184). HbA1c and fasting glucose were progressively lower across the low-, intermediate-, and target-TIR groups.

Importantly, a statistically significant time × TIR group interaction was identified only for fasting glucose (F = 3.793, *p* = 0.023, partial η^2^ = 0.014), indicating that the magnitude of the 12-week change in fasting glucose differed among the TIR groups. The low-TIR group showed the greatest mean reduction in fasting glucose (−28.69 ± 91.15 mg/dL), followed by the intermediate-TIR (−18.37 ± 59.94 mg/dL) and target-TIR groups (−10.59 ± 37.52 mg/dL). In contrast, the time × TIR interactions were not significant for HbA1c (*p* = 0.089), total cholesterol (*p* = 0.402), triglycerides (*p* = 0.540), HDL cholesterol (*p* = 0.097), or LDL cholesterol (*p* = 0.184). Thus, although several glycemic and lipid parameters changed over time or differed according to TIR category, fasting glucose was the only parameter for which the temporal change differed significantly among the three TIR groups.

Supplementary Figure S2 also illustrates the changes in glycemic and lipid parameters from baseline to 12 weeks across the three TIR groups. HbA1c decreased consistently in all groups, from 9.27% to 8.41% in the Low-TIR group, from 8.26% to 7.64% in the Intermediate-TIR group, and from 7.56% to 7.02% in the Target-TIR group (Figure S2a). Similarly, fasting glucose decreased in all three groups, with the largest absolute reduction observed in the Low-TIR group (187.95 to 159.26 mg/dL), followed by the Intermediate-TIR group (156.08 to 137.71 mg/dL) and the Target-TIR group (137.29 to 126.70 mg/dL) (Figure S2b). These patterns were consistent with the significant time effects observed for both glycemic parameters, while a significant time × TIR group interaction was observed only for fasting glucose.

Changes in lipid parameters showed more heterogeneous patterns across the TIR groups (Figure S2c–f). Total cholesterol remained nearly unchanged in the Low-TIR group (150.81 to 151.19 mg/dL) but decreased in the Intermediate-TIR (149.98 to 146.13 mg/dL) and Target-TIR groups (149.90 to 145.67 mg/dL) (Figure S2c). Triglyceride levels decreased in all three groups, with the largest absolute reduction observed in the Low-TIR group, followed by the Target-TIR and Intermediate-TIR groups (Figure S2d). HDL cholesterol increased in the Low-TIR and Target-TIR groups but remained essentially unchanged in the Intermediate-TIR group (Figure S2e). LDL cholesterol showed a slight increase in the Low-TIR group, whereas decreases were observed in the Intermediate-TIR and Target-TIR groups, with the greatest reduction occurring in the Target-TIR group (Figure S2f). Despite these numerical differences in the trajectories of lipid parameters, none of the time × TIR group interactions for total cholesterol, triglycerides, HDL cholesterol, or LDL cholesterol reached statistical significance, indicating that the 12-week changes in lipid parameters did not differ significantly among the three TIR groups.

### 3.7. Multivariable Analysis of the Association Between TIR Group and Lipid Parameters

Multivariable regression analyses were additionally performed to assess whether the associations between TIR group and lipid parameters persisted after accounting for baseline differences in glycemic control (Supplementary Table S2). The models were adjusted for age, body mass index, baseline HbA1c, and baseline fasting glucose. Compared with the Low-TIR group, the Intermediate-TIR group was significantly associated with lower total cholesterol (B = −8.508, *p* = 0.031) and LDL cholesterol (B = −6.697, *p* = 0.043). Similarly, the Target-TIR group was associated with lower total cholesterol (B = −11.176, *p* = 0.005) and LDL cholesterol (B = −10.290, *p* = 0.002) after multivariable adjustment. In contrast, neither the Intermediate- nor Target-TIR group showed significant adjusted associations with triglycerides or HDL cholesterol (all *p* > 0.05). These findings indicate that the associations of higher TIR categories with lower total and LDL cholesterol remained significant after adjustment for age, BMI, and baseline glycemic status, whereas the corresponding associations with triglycerides and HDL cholesterol were not statistically significant.

**Table 6:** Glycemic and Lipid Parameter Changes After 12-Week CGM Application Stratified by Time in Range (TIR) Achievement (*n* = 542).

Variable	Low (TIR < 50%, *n* = 111)				Intermediate (TIR 50–70%, *n* = 164)				Target (TIR > 70%, *n* = 267)			
	Baseline, Mean ± SD	12 Weeks, Mean ± SD	Mean Change	*p*-Value	Baseline, Mean ± SD	12 Weeks, Mean ± SD	Mean Change	*p*-Value	Baseline, Mean ± SD	12 Weeks, Mean ± SD	Mean Change	*p*-Value
HbA1C (%)	9.27 ± 1.72	8.41± 1.25	−0.86	<0.001	8.26 ± 1.14	7.64± 0.93	−0.62	<0.001	7.56 ± 1.01	7.02± 0.80	−0.54	<0.001
Fasting glucose (mg/dL)	187.95 ± 79.33	159.26 ± 62.04	−28.69	0.001	156.08 ± 57.01	137.71 ± 35.46	−18.37	<0.001	137.29 ± 38.55	126.70 ± 28.20	−10.59	<0.001
Total cholesterol (mg/dL)	150.81 ± 37.99	151.19 ± 37.19	0.38	0.904	149.98 ± 36.86	146.13 ± 33.55	−3.85	0.133	149.90 ± 39.27	145.67 ± 33.88	−4.23	0.020
Triglyceride (mg/dL)	163.28 ± 113.99	147.85 ± 89.32	−15.43	0.113	142.99 ± 89.52	139.18 ± 85.29	−3.81	0.560	136.99 ± 106.35	125.87 ± 99.25	−11.12	0.038
HDL	45.14 ± 13.26	46.20 ± 12.75	1.06	0.197	45.40 ± 11.73	45.29 ± 12.37	−0.11	0.826	47.50 ± 11.55	48.84 ± 11.88	1.34	0.001
LDL	74.42 ± 31.31	75.34 ± 29.18	0.92	0.718	75.98 ± 29.11	73.40 ± 25.40	−2.58	0.230	74.77 ± 30.86	70.29 ± 26.38	−4.48	0.004

## 4. Discussion

This study evaluated metabolic changes in 542 patients with T2DM following 12 weeks of CGM-guided management in a real-world clinical setting. Consistent with previous studies [10,25], CGM adoption was associated with statistically significant reductions in mean HbA1c (8.12% to 7.49%) and fasting plasma glucose (153.35 mg/dL to 136.70 mg/dL), alongside favorable shifts in circulating lipid fractions, including decreases in total cholesterol, triglycerides, and LDL cholesterol, and an increase in HDL cholesterol.

A central finding of this investigation emerged from the stratification analysis across TIR achievement levels. Although patients across all TIR levels (TIR < 50%, 50–70%, and >70%) achieved significant reductions in HbA1c and fasting glucose, statistically significant improvements across all serum lipid fractions were observed in patients achieving the target TIR threshold (>70%), which was similar to the results of previous study [26,27]. However, this pattern should be interpreted cautiously, as the unequal sample sizes across the TIR strata, particularly the larger sample size of the Target-TIR group, may have influenced the statistical power to detect within-group changes. Moreover, because no formal multiplicity adjustment was applied, the within-group findings were considered exploratory and interpreted alongside the repeated-measures ANOVA and adjusted multivariable analyses. Therefore, the statistical significance observed in the Target-TIR group should not be interpreted as evidence that lipid improvements occur exclusively at this TIR level or as evidence of a TIR-specific biological effect.

Improved glycemic control during the 12-week period may have contributed to favorable changes in lipid metabolism, potentially through the alleviation of glucotoxicity and associated metabolic disturbances [28]. However, the present data do not permit determination of the underlying biological mechanisms or attribution of the observed lipid changes specifically to CGM use or TIR attainment. Other factors, including regression to the mean, increased treatment adherence or behavioral modification associated with study participation (i.e., a Hawthorne effect), and unrecorded changes in glucose- or lipid-lowering medications, may also have contributed to the observed findings. Therefore, the modest lipid changes observed in the Target-TIR group should be regarded as associations rather than evidence of a direct mechanistic effect of achieving TIR >70%.

These real-world clinical findings align with recommendations from international diabetes consensus guidelines and clinical trial meta-analyses. The 2019 International Consensus on Time in Range, supported by the ADA and EASD, established TIR >70% as a primary target goal for non-pregnant adults with diabetes [29]. Meta-analyses of randomized controlled trials demonstrate that CGM use consistently reduces HbA1c, increases TIR, and reduces glycemic variability [30]. The findings of this study extend these clinical trial observations to real-world Asian populations managed in outpatient settings.

Our findings also align with observational studies linking TIR to vascular complication risks. Lu et al. demonstrated that lower TIR independently correlates with an increased prevalence of diabetic retinopathy, carotid intima-media thickness progression, and overall cardiovascular mortality [31]. Similarly, Yoo et al. identified low TIR as an independent risk factor for albuminuria [9], while Mayeda et al. reported an association between lower TIR and diabetic peripheral neuropathy symptoms [32]. By demonstrating that achieving TIR >70% is associated with favorable changes in systemic lipid fractions, our study suggests that favorable lipid modulation may serve as a contributing pathway through which high TIR protects against macro-vascular disease.

The observed variations in triglyceride responsiveness across demographic subgroups offer practical considerations for personalized diabetes management. Male participants and younger adults (18–39 years) achieved greater triglyceride reductions compared to female participants and older age groups [33,34]. The pronounced lipid response in younger adults may reflect higher baseline metabolic plasticity, greater capacity for insulin sensitivity recovery, or distinct dietary adaptations following direct visual feedback from real-time glucose displays.

Conversely, the elevated triglyceride response observed in underweight participants (BMI < 18.5 kg/m^2^) underscores the heterogeneity of T2DM phenotypes. Underweight individuals with diabetes frequently exhibit distinct pathophysiological features, including severe beta-cell secretory impairment or lean insulin resistance, rather than classic metabolic syndrome [35]. In these populations, glycemic instability may stem from insulin deficiency rather than overnutrition, requiring tailored clinical management strategies.

Rather than adjusting therapy based solely on quarterly HbA1c values, clinicians can leverage detailed CGM data to optimize management strategies. Real-time trend arrows and glycemic pattern visibility empower patients to make timely lifestyle modifications, including dietary adjustments and post-meal physical activity. Furthermore, integrating TIR targets into routine workflows establishes a comprehensive approach to cardiometabolic risk reduction.

Several limitations should be considered when interpreting these results. First, the retrospective, single-center pre–post study design precludes definitive causal inferences regarding the independent effect of CGM on lipid changes relative to prospective randomized controlled trials and may limit the generalizability of the findings to other populations, healthcare settings, and CGM systems.

Second, the clinical relevance of the observed lipid changes should be interpreted cautiously because baseline total cholesterol and LDL levels were already relatively well controlled, and the absolute magnitude of further lipid reduction was modest. The present study did not evaluate achievement of guideline-directed LDL targets or perform subgroup analyses among participants with baseline dyslipidemia.

Furthermore, detailed information on changes in concomitant pharmacological therapies, including statins, SGLT2 inhibitors, and GLP-1 receptor agonists, as well as dietary or exercise interventions during the 12-week period, was not consistently available. Therefore, residual confounding by co-interventions cannot be excluded, and the observed lipid changes should not be attributed solely to CGM use or TIR attainment.

Another limitation is that scanning frequency data were not analyzed in this study. Because scan frequency in intermittently scanned CGM reflects the degree of patient engagement, active self-monitoring, and motivation, unmeasured differences in scanning behavior may have confounded the associations between TIR and metabolic outcomes.

This study was also limited by the absence of detailed longitudinal information on glycemic patterns during the 12-week CGM period. Glycemic changes were primarily assessed using pre- and post-intervention measurements, precluding a detailed evaluation of dynamic glycemic trajectories during CGM use. Consequently, we could not determine whether progressively improving, stable, or fluctuating glycemic patterns were differentially associated with lipid changes.

In addition, prior CGM experience was not systematically documented; therefore, the cohort may have included both CGM-naive individuals and patients with previous CGM exposure. Because prior CGM use may influence self-management behavior, adherence, and metabolic responses, residual confounding related to previous CGM experience cannot be excluded.

A further limitation is that no predefined allowable time window was established for baseline or follow-up laboratory measurements. Because blood sampling was performed according to routine clinical schedules, the exact timing of laboratory assessments relative to CGM initiation and the intended 12-week follow-up may have varied among participants, potentially introducing temporal heterogeneity into the observed metabolic changes.

Future multicenter prospective studies with standardized follow-up protocols and comprehensive assessment of medications, behavioral factors, CGM engagement, and prior CGM exposure are needed to confirm these findings in more diverse populations.

## 5. Conclusions

This real-world retrospective study demonstrated that continuous glucose monitoring-derived Time in Range (TIR) was associated with glycemic and selected lipid parameters in adults with type 2 diabetes mellitus. Twelve weeks of CGM-guided management yielded statistically significant reductions in HbA1c and fasting plasma glucose, alongside modest modifications in circulating lipid parameters across the study cohort.

These findings should be interpreted cautiously and not as evidence of causal effects; nevertheless, they suggest that TIR may have potential as a useful complementary metric to HbA1c for reflecting broader glycemic and metabolic status in clinical practice. Further studies are needed to clarify the relationship between TIR and lipid metabolism and to determine its potential role in comprehensive diabetes management.

## Figures and Tables

**Table 1:** Single-arm Pre–Post Study Design.

**Group**	**Pre-test**	**→**	**Interventions**	**→**	**Post-test**
All participants	HbA1c, Fasting Plasma Glucose, Total Cholesterol, Triglycerides, HDL-C, LDL-C		Continuous monitoring via factory-calibrated CGM system		HbA1c, Fasting Plasma Glucose, Total Cholesterol, Triglycerides, HDL-C, LDL-C

**Table 2:** Baseline Sociodemographic and Health-Related Characteristics of Study Subjects (*n* = 542).

Variable	*n* (%)	Mean ± SD
Sex		
Male	344 (63.5)	
Female	198 (36.5)	
Age ^1^		60.41 ± 11.17
18–39	22 (4.1)	
40–64	305 (56.3)	
≥65	215 (39.6)	
BMI ^2^		25.25 ± 3.72
Low weight (<18.5)	10 (1.8)	
Normal (18.5–24.9)	258 (47.6)	
Obesity (≥25)	274 (50.6)	
Smoking status		
Never smoker	435 (80.3)	
Current smoker	49 (9.0)	
Former smoker	58 (10.7)	
Alcohol consumption		
Non-drinker	482 (88.9)	
Current drinker	56 (10.3)	
Former drinker	4 (0.8)	

SD: standard deviation. ^1^ Units expressed as years. ^2^ Units expressed as kg/m^2^.

**Table 3:** CGM use Characteristics of Study Subjects (*n* = 542).

Variable	Mean ± SD	Consensus Target
Average fasting glucose (mg/dL)	167.99 ± 37.57	-
Glucose Management Indicator (%)	7.47 ± 3.25	<7.0%
Coefficient of Variation (%)	29.93 ± 7.21	≤36.0%
CGM sensor active time (%)	84.93 ± 18.35	≥70.0%
TAR 2 (>250 mg/dL, %)	10.85 ± 14.62	<5.0%
TAR 1 (181–250 mg/dL, %)	23.01 ± 11.37	<25.0%
TIR (70–180 mg/dL, %)	65.30 ± 21.87	>70.0%
TBR 1 (54–69 mg/dL, %)	0.72 ± 2.14	<4.0%
TBR 2 (<54 mg/dL, %)	0.13 ± 1.60	<1.0%

**Table 4:** Changes in Glycemic and Lipid Parameters Before and After CGM Intervention (*n* = 542).

Variable	Mean ± SD		Mean Change	t	*p*-Value
	Baseline	Post-CGM			
HbA1C (%)	8.12 ± 1.39	7.49 ± 1.09	−0.63	11.038	<0.001
Fasting glucose (mg/dL)	153.35 ± 58.01	136.70 ± 41.31	−16.65	6.540	<0.001
Total cholesterol (mg/dL)	150.11 ± 38.23	146.94 ± 34.49	−3.17	2.369	0.018
Triglyceride (mg/dL)	144.19 ± 103.53	134.39 ± 93.47	−9.80	2.552	0.011
HDL (mg/dL)	46.38 ± 12.00	47.23 ± 12.30	0.85	−2.862	0.004
LDL (mg/dL)	75.07 ± 30.39	72.27 ± 26.72	−2.80	2.499	0.013

**Table 5:** Change in triglycerides according to sociodemographic and health-related characteristics (*n* = 542).

Variable	Triglyceride Variation	t, F	*p*-Value
	Mean ± SD		
Sex		−2.474	0.014
Male	−16.27 ± 99.58		
Female	1.46 ± 66.79		
Age ^1^		4.000	0.019
18–39 (a)	−61.82 ± 144.54	a > b, a > c	
40–64 (b)	−6.36 ± 93.92		
≥65 (c)	−9.35 ± 72.87		
BMI ^2^		3.666	0.026
Low weight (<18.5) (a)	52.40 ± 90.74	b > a	
Normal (18.5–24.9) (b)	−17.05 ± 83.35		
Obesity (≥25) (c)	−5.23 ± 93.81		
Smoking status		0.457	0.633
Never smoker	−11.60 ± 78.51		
Current smoker	−1.25 ± 138.30		
Former smoker	−3.48 ± 112.34		
Alcohol consumption		0.013	0.987
Non-drinker	−9.98 ± 84.74		
Current drinker	−8.64 ± 125.26		
Former drinker	−4.50 ± 30.95		

SD: standard deviation. ^1^ Units expressed as years. ^2^ Units expressed as kg/m^2^.

## Data Availability

The data presented in this study are available on request from the corresponding author.

## Supplementary Materials

The following supporting information can be downloaded at: https://www.mdpi.com/article/10.3390/life16101667/s1, Table S1. Repeated Measures ANOVA Results for the Impact of TIR Group on Changes of Glycemic and Lipid Parameters (*n* = 542); Table S2. Multivariable Linear Regression Analysis for Association Between TIR Group and 12-Week Lipid Parameters After Adjustment for Baseline Clinical and Glycemic Factors (*n* = 542); Figure S1 Relationship between CGM-derived Time in Range (TIR) and change in triglyceride levels. The scatter plot shows individual observations, and the solid line represents the fitted cubic regression curve. No clear nonlinear pattern was observed across the range of TIR values; Figure S2 Changes in Glycemic and Lipid Parameters from Baseline to 12 Weeks According to Time in Range (TIR) Group (a) HbA1c, (b) fasting glucose, (c) total cholesterol, (d) triglycerides, (e) HDL cholesterol, and (f) LDL cholesterol.
