# Supplementary material for: Time in Range as a Marker of Concurrent Glycemic and Lipid Improvement in Adults with Type 2 Diabetes: A Real-World Retrospective Pre–Post Study
Source: Life (Basel). 2026 Oct 5;16(10):1667. doi: 10.3390/life16101667 (PMC13705905; doi:10.3390/life16101667)
Supplement: Supplementary file 1 [file life-16-01667-s001.zip › life-4562195-supplementary.pdf]

Supplementary Materials

Table S1. Repeated Measures ANOVA Results for the Impact of TIR Group on Changes of Glycemic and Lipid Parameters (*n* = 542).

Variable	TIR Group	Baseline	12 Weeks	Change	Time Effect	TIR Effect	Time × TIR (Interaction)	Partial η² (Interaction)
		Mean ± SD			F (*p*)			
Changes in HbA1C (%)	Low [a]	9.27 ± 1.72	8.41± 1.25	−0.86 ± 1.98	123.820 (<0.001)	126.112 (<0.001)	2.425 (0.089)	0.009
	Intermediate [b]	8.26 ± 1.14	7.64± 0.93	−0.62 ± 1.21	a>b, a>c, b>c			
	Target [c]	7.56 ± 1.01	7.02± 0.80	−0.54 ± 0.99				
Changes in Fasting glucose (mg/dL)	Low [a]	187.95 ± 79.33	159.26 ± 62.04	−28.69 ± 91.15	50.693 (<0.001)	48.416 (<0.001)	3.793 (0.023)	0.014
	Intermediate [b]	156.08 ± 57.01	137.71 ± 35.46	−18.37 ± 59.94	a>b, a>c, b>c			
	Target [c]	137.29 ± 38.55	126.70 ± 28.20	−10.59 ± 37.52				
Changes in Total cholesterol (mg/dL)	Low [a]	150.81 ± 37.99	151.19 ± 37.19	0.38 ± 32.86	3.239 (0.072)	0.397 (0.673)	0.913 (0.402)	0.003
	Intermediate [b]	149.98 ± 36.86	146.13 ± 33.55	−3.85 ± 32.61				
	Target [c]	149.90 ± 39.27	145.67 ± 33.88	−4.23 ± 29.47				
Changes in Triglyceride (mg/dL)	Low [a]	163.28 ± 113.99	147.85 ± 89.32	−15.43 ± 101.87	6.121 (0.014)	3.025 (0.049)	0.617 (0.540)	0.002
	Intermediate [b]	142.99 ± 89.52	139.18 ± 85.29	−3.81 ± 83.72				
	Target [c]	136.99 ± 106.35	125.87 ± 99.25	−11.12 ± 87.28				
Changes in HDL	Low [a]	45.14 ± 13.26	46.20 ± 12.75	1.06 ± 8.62	5.937 (0.015)	3.697 (0.025)	2.344 (0.097)	0.009
	Intermediate [b]	45.40 ± 11.73	45.29 ± 12.37	−0.11 ± 6.39	b<c			
	Target [c]	47.50 ± 11.55	48.84 ± 11.88	1.34 ± 6.29				
Changes in LDL	Low [a]	74.42 ± 31.31	75.34 ± 29.18	0.92 ± 26.75	2.946 (0.087)	0.523 (0.0.184)	1.696 (0.184)	0.006
	Intermediate [b]	75.98 ± 29.11	73.40 ± 25.40	−2.57 ± 27.36				
	Target [c]	74.77 ± 30.86	70.29 ± 26.38	−4.49 ± 24.92				

TIR group: [a] Low (TIR < 50%); [b] Intermediate (TIR 50–70%); [c] Target (TIR > 70%).

Table S2. Multivariable Linear Regression Analysis for Association Between TIR Group and 12-Week Lipid Parameters After Adjustment for Baseline Clinical and Glycemic Factors (*n* = 542).

Variable		B	t	*p*	VIF [1]	Model Fit [2]		
						F	*p*	adj. R^2
Total cholesterol (mg/dL)	Low (TIR < 50)	Reference				4.276	0.000 [c]	0.035
	Intermediate (TIR 50–70)	−8.508	−2.167	0.031	1.883			
	Target (TIR > 70)	−11.176	−2.821	0.005	2.271			
Triglyceride (mg/dL)	Low (TIR < 50)	Reference				6.289	0.000 [c]	0.055
	Intermediate (TIR 50–70)	−1.387	−0.124	0.901	1.883			
	Target (TIR > 70)	−16.074	−1.429	0.153	2.271			
HDL	Low (TIR < 50)	Reference				2.526	0.020 [c]	0.017
	Intermediate (TIR 50–70)	−0.944	−1.079	0.281	1.883			
	Target (TIR > 70)	0.544	0.616	0.538	2.271			
LDL	Low (TIR < 50)	Reference				3.692	0.001 [c]	0.029
	Intermediate (TIR 50–70)	−6.697	−2.030	0.043	1.883			
	Target (TIR > 70)	−10.290	−3.092	0.002	2.271			

[1] VIF: Variance Inflation Factor; [2] Model Fit: adjusted for baseline characteristics (age, body mass index, HbA1C and fasting glucose). [c] *p*-value for the overall F-test of the multivariable linear regression model.

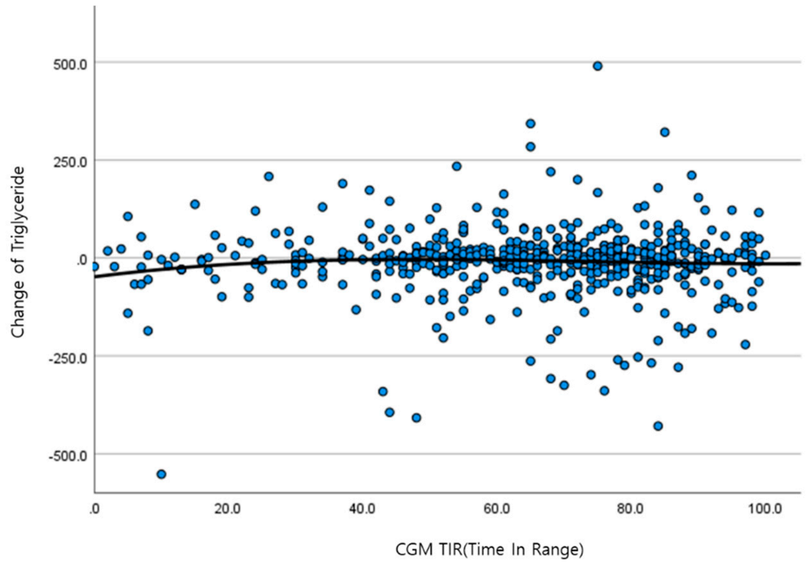

Figure S1. Relationship between CGM-derived Time in Range (TIR) and change in triglyceride levels. The scatter plot shows individual observations, and the solid line represents the fitted cubic regression curve. No clear nonlinear pattern was observed across the range of TIR values.

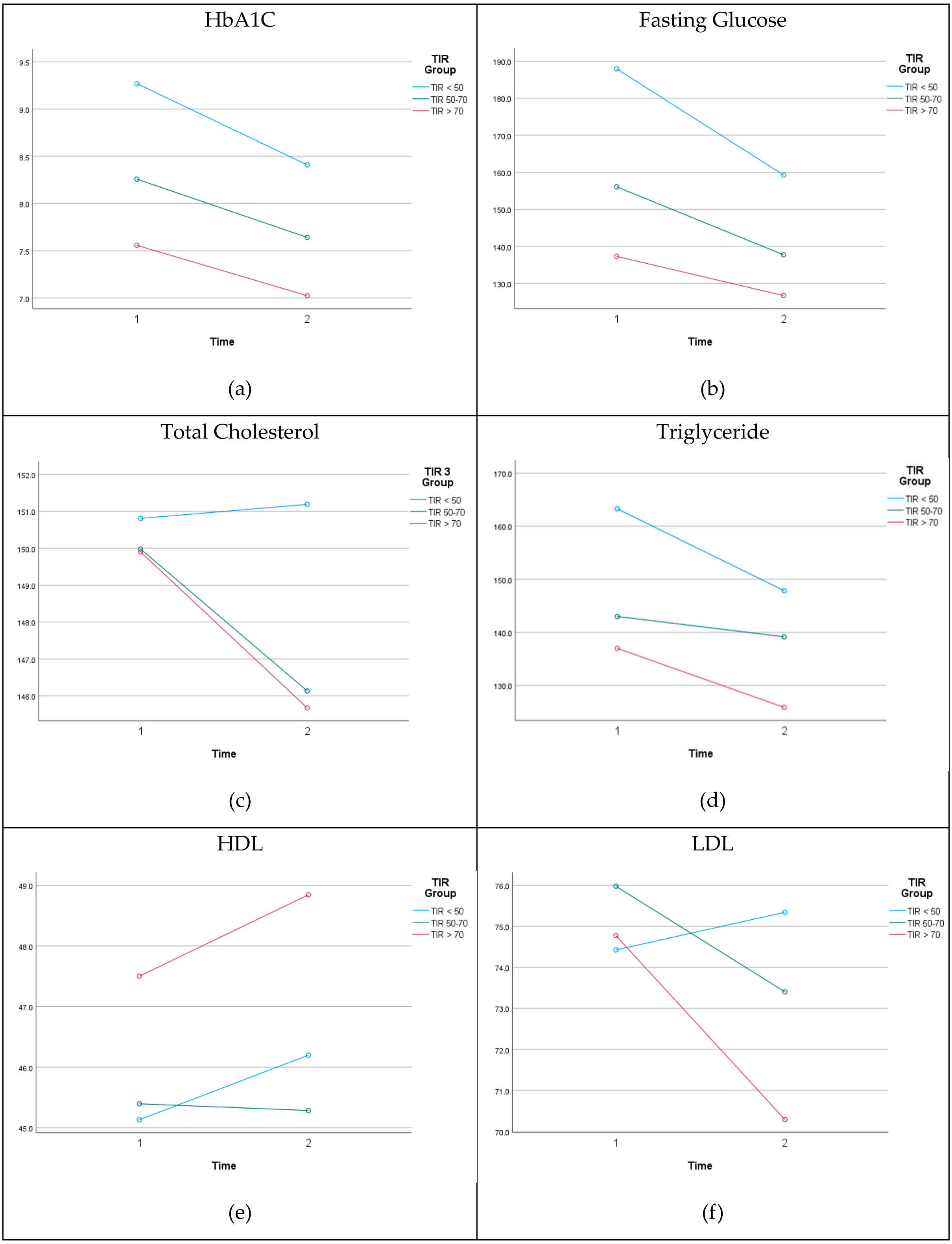

Figure S2. Changes in Glycemic and Lipid Parameters from Baseline to 12 Weeks According to Time in Range (TIR) Group. (**a**) HbA1c, (**b**) fasting glucose, (**c**) total cholesterol, (**d**) triglycerides, (**e**) HDL cholesterol, and (**f**) LDL cholesterol.
